# Pretreated Green Pea Flour as Wheat Flour Substitutes in Composite Bread Making

**DOI:** 10.3390/foods12122284

**Published:** 2023-06-06

**Authors:** Oscar Moreno-Araiza, Fatma Boukid, Xinying Suo, Shihao Wang, Elena Vittadini

**Affiliations:** 1 School of Biosciences and Veterinary Medicine, University of Camerino, Via Gentile III da Varano, 62032 Macerata, Italy; oscar.morenoaraiza@unicam.it (O.M.-A.); xinying.suo@unicam.it (X.S.); shihao.wang@studenti.unicam.it (S.W.); 2 ClonBio Group Ltd., D02 XE61 Dublin, Ireland; fboukid@clonbioeng.com

**Keywords:** legumes, pre-gelatinization, rheology, shelf life, texture, starch digestibility

## Abstract

The present study aimed to assess the impact of substituting wheat flour with three different pretreated green pea flour at different addition levels (10–50%) on fresh bread quality during a 7-day storage period. Dough and bread enriched with conventionally milled (C), pre-cooked (P), and soaked under-pressure-steamed (N) green pea flour were evaluated for their rheological, nutritional, and technological features. Compared to wheat flour, legumes had lower viscosity but higher water absorption, development time, and lower retrogradation. Bread made with C10 and P10 showed similar specific volume, cohesiveness, and firmness to the control, whereas addition levels beyond 10% decreased specific volume and increased firmness. During storage, incorporating legume flour (10%) delayed staling. Composite bread increased proteins and fiber. C30 had the lowest rate of starch digestibility, while pre-heated flour increased starch digestibility. In conclusion, P and N can be considered valuable ingredients for making soft and stable bread.

## 1. Introduction

Bread is a staple food worldwide, and it is chiefly prepared from refined wheat flour. There is an increasing interest in fortified bread for health, sustainability, and marketing [1]. Composite flour bread is made by partially replacing wheat flour with various types of plant ingredients. Legumes are particularly attractive ingredients for food manufacturers due to their nutritional features [2,3]. They are rich sources of proteins, fibers, phytochemicals, minerals, and vitamins [4]. In bread, the addition of legume flour has been recognized as a valid strategy to improve their nutritional quality, particularly proteins, by compensating for the deficiency of some amino acids, such as lysine [5]. In addition, legumes are known as sustainable crops owing to their ability to fix nitrogen. These attributes make legumes a competitive, sustainable ingredient for making innovative bakeries. Nevertheless, substituting wheat flour with legume flour may cause technological challenges due to its different functional properties compared to wheat gluten. Additionally, the presence of off-favor compounds can limit consumer acceptability of legume-enriched bread [6].

Therefore, several pretreatment strategies were applied to overcome these limitations. Milling and size fractionation are conventional processes affecting the physicochemical properties of legume flour [4,7]. For instance, lentil flour having different particle sizes (coarse, medium, fine, and extra-fine) induced significant effects on dough properties [8]. Germination of legumes reduced antinutritional factors (e.g., tannin, oligosaccharides, and phytic acid) and increased protein availability [9]. Consequently, the incorporation of germinated pea flour (50% addition level) increased protein and fiber contents and improved the volume and texture of bread [10]. Fermentation also shows momentum for improving the nutritional and organoleptic properties of legumes [11,12]. Substituting wheat flour with fermented legume flour improved the nutritional quality (increased protein digestibility and reduced predicted glycemic index) of the composite bread [13]. Heat treatments, such as roasting, steaming, and extrusion, can reduce the antinutritional compounds (e.g., tannins and phytic acid) and improve the flavor profile of legumes [14,15,16]. For instance, the use of roasted yellow pea flour (10%) improved bread flavor and increased the specific volume [14]. Extruded mung and pea flour showed interesting functional properties due to starch gelatinization that impacted the dough water absorption and stability [17,18]. In recent years, innovative processing technologies, such as high hydrostatic pressure and ultrasound, have been applied to improve the functionality of legume flour [19]. High-pressure processing (350 MPa, 10 min) resulted in starch gelatinization and protein denaturation, leading to improved viscoelastic properties of chickpea- and pea-based doughs [20]. Ultrasound lupin flour had reduced bitterness, and its inclusion (10–20%) in bread increased the specific volume and decreased crumb firmness [21].

As such, the properties of pretreated legume flour differ based on the type and severity of the pretreatment. For this reason, the present study went deeper, investigating the performance of pretreated green pea flour using conventional and emerging physical treatments. Conventional flour obtained using traditional milling, precooked flour using hydrothermal treatment, drying, and milling, and a “new generation” flour obtained by water-soaking under pressure, steaming, drying, and milling were used as wheat flour substitutes. The objective was to evaluate the effect of pretreated green pea flour addition (at different levels from 10 to 50%) on the nutritional and technological properties of fresh composite bread during storage. To achieve this goal, the experimental design was divided into three subjective phases:

- investigation of pasting properties of individual untreated and pretreated green pea flour in addition to dough mixing properties of selected composite flour in comparison with wheat flour;
- effect of pretreated green pea flour on fresh bread technological quality and during storage. In this section, a full factorial design was considered in association with multivariate analysis for a better understanding of the impact of each studied factor (level of the addition, type of pretreatment, storage period, and their interaction);
- effect on nutritional quality with a focus on starch digestibility in the product with industrial relevance.

## 2. Materials and Methods

### 2.1. Wheat and Green Pea Flour

Wheat flour (W; Corradini Mill, Mogliano [MC], Italy) and three types of commercial green pea flour (Martino Rossi SpA, Cremona, Italy) were considered in this study (Table S1). Green pea flour samples were labeled as conventional (C; obtained by traditional milling), precooked (P; obtained by hydrothermal treatment, drying, and milling), or water-soaked (N; obtained by water-soaking under pressure, steaming, drying, and milling).

### 2.2. Dough Rheological Properties

Viscoamylograph analysis of flour was determined using a Micro Visco-Amylo-Graph Brabender Instruments (Duisburg, Germany). Different types of flour (9 g) were suspended in distilled water (60 mL) forming flour slurries, heated from 30 °C to 98 °C at a heating rate of 7.5 °C/min, held at 98 °C for 2 min, cooled to 30 °C, and held for 4 min. The viscosity values at the beginning (initial viscosity, IV), at the peak (peak viscosity, PV), at the end of the holding period at 98 °C (minimum viscosity, MV), and at the end of the cooling phase at 30 °C (cooling maximum viscosity, CMV) were recorded and expressed in arbitrary Brabender units (BU). The differences between PV and MV and between CMV and MV were defined as a “breakdown” and a “setback”, respectively. The analysis was conducted in triplicate.

Farinograph curves were obtained using a Brabender instrument (Brabender OHG, Duisburg, Germany). The analysis was made with 50% blends of pretreated green pea flour and wheat flour to evaluate the legume flour’s effect on wheat flour dough quality. The 50% replacement level was selected to perform the farinograph experiments to understand the potential changes in dough behavior at the maximum level of the addition and compared to wheat flour. The farinograph parameters were measured according to AACC Approved Method 54-21.02 [22]. Three determinations were performed for each sample.

### 2.3. Bread Formulation, Production, and Storage

The bread was produced following a standard white bread recipe (AACC method 10-10.03) [22]. For bread preparation, the ingredients [salt (Don Jerez^®^, Milan, Italy), sugar (Dolciando^®^, Milan, Italy), yeast (Tre Mulini^®^, Milan, Italy), and olive oil (La Badia^®^, Parma, Italy)] were bought from a local supermarket. Table 1 illustrates the formulations of control (100% wheat flour) and bread enriched with pre-treated green pea flour (C, P, and N) at different levels (10–50%). Using the farinograph, the optimal water was determined for each bread formulation to obtain a dough with a consistency of 500 BU. The bread was made using a bread maker machine [IMETEC^®^ ZERO-GLU, Azzano San Paolo (BG), Italy]. First, water and yeast were mixed for 2 min, followed by flour, sugar, olive oil, and then salt (after 2 min of mixing). The selected baking program (number 14, maximum browning level) consisted of the dough mixing phase (22 min), levitation (30 °C, 82 min), and baking (210 °C, 117 min). After baking, the bread was left to cool down at room temperature. Then, each bread loaf was placed into a polyethylene bag sprayed with 2 mL of ethyl alcohol (to prevent mold growth) and stored at 25 °C for a week. Each bread formulation was performed in triplicate.

### 2.4. Technological Properties of Fresh and Stored Bread

The volume of the fresh bread was measured using 3 different bread loaves for each bread type using a standard rapeseed displacement method according to AACC method 10–05.01 [22]. Specific volume was calculated as the ratio of volume to weight.

Moisture and texture were determined on fresh bread and after 1, 3, and 7 days. Crumb moisture content was measured from weight loss at 105 °C until constant weight, as described in the AACC method 44-15.02 [22]. Bread crumb texture was measured using a Texture Analyzer (TA1, AMETEK, Inc., Berwyn, PA, USA). Crumb bread cubes (2.5 × 2.5 × 2.5 cm; 12 from each loaf) were extracted from the center of each bread loaf and were subjected to a double-compression cycle using a 36 mm diameter cylindrical probe to a 40% deformation at a test speed of 1 mm/s, with a 5 s wait between compression cycles. Textural parameters considered were hardness (maximum force during the first compression) and cohesiveness (ratio of the area of the two compression cycles). At least 12 repetitions were made for each sample.

### 2.5. Bread Nutritional Quality

The nutritional facts for fresh bread were computed based on data provided on commercial product labels or technical sheets provided by the manufacturers. The energy value was calculated using the energy factors provided in EU Regulation n° 1169/2011 [23], in which carbohydrates (excluding polyols), protein, fats, and fibers accounted for 4 kcal/g, 4 kcal/g, 9 kcal/g, and 2 kcal/g, respectively.

In vitro, the starch digestibility of selected fresh bread was evaluated [24]. In brief, rapidly available glucose (RAG) was determined as the amount of glucose released during the first 20 min (G20 = RAG), and slowly available glucose (SAG) was determined as the glucose released between 20 min and 120 of enzyme hydrolysis (D120-D20). The final determination of glucose was measured by a D-glucose assay kit in GOPOD format (Megazyme International, Wicklow, Ireland). The starch fractions were calculated as following parameters: rapidly digestible starch (RDS) = 0.9 × (G20 − FGS); slowly digestible starch (SDS) + 0.9 × (G120 − G20); resistant starch (RS); 0.9 × (TG − G120); total starch (TS) 0.9 × (TG − FSG). The starch digestion index (SDI) was calculated by the following formula: (RDS/TS) × 100%. Three determinations were performed for each sample.

Resistant starch (RS) and total starch of bread samples were determined using the Resistant Starch Assay kit (Megazyme International, Wicklow, Ireland) [25]. The units of RDS, SDS, and RS were expressed in grams of starch per 100 g of total starch (% of total starch on a wet basis).

### 2.6. Statistical Analysis

Multivariate analysis of variance (MANOVA) was used to determine the effect of the type of pretreatment (PT), level of addition (LA), and storage (S) on the bread properties. MANOVA was performed based on fixed factors using Pillai’s trace test. The percentages of total variations were computed to determine the contribution of the factors (PT, LA, and S) and their interactions in the variance of each parameter. The percentage of the total variation was computed to explain the variance of each parameter as a function of the sum squares of the main factors and their interaction. Significant differences among the mean values were calculated using Duncan’s test. A principal component analysis (PCA) was performed based on the correlation matrix. All experimental data were statistically analyzed using SPSS version 19.0 (SPSS Inc., Chicago, IL, USA).

## 3. Results and Discussion

### 3.1. Rheological Properties

**Figure 1:**
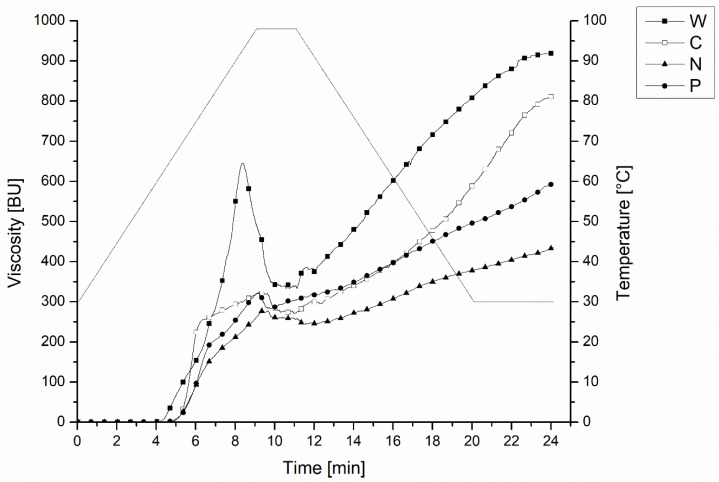
Pasting profiles of green pea flour with different pre-treatments, control wheat flour (W), conventional (C), soaked, under-pressure-steamed (N), and pre-cooked (P).

The pasting properties of the flour used for preparing the bread are reported in Table 2. As illustrated in Figure 1, all viscosity values were found to be higher in wheat flour compared to legumes. This can be due to the high protein content in legumes limiting starch swelling, as reported in previous studies. Statistically, the initial viscosity W, P, and N were similar. P showed significantly lower viscosity than W but was statistically similar to N. P and N were expected to have higher initial viscosity because the pretreatments included a hydrothermal step that was expected to have an important impact on starch structure favoring starch gelatinization, while in C, the starch remained in its native form [26]. Besides starch, pretreatment might have caused protein denaturation and fiber modification, thus affecting protein–fiber structures and water redistribution among solids [27,28], which affected the dynamic inside the dough matrix [14,29]. The important viscosity increase observed in P and N above 69 °C suggested the presence of residual ungelatinized starch in the flour. Green pea flour gelatinization started at higher temperatures compared to W, but pretreatment did not impact the temperature at the beginning of gelatinization, suggesting that only partial starch gelatinization occurred during the pretreatment. Similarly, the peak of viscosity was the highest in W due to higher starch and lower protein contents compared to legume flour [30]. Pretreatment did not impact the peak of viscosity, and it required a higher temperature to gelatinize compared to W. Low peak viscosity was also observed in pre-gelatinized carioca bean flour [31]. During the cooling, the maximum viscosity was found to be the highest in W, indicating a strong tendency toward starch retrogradation. However, legume flour showed less tendency for retrogradation, where N had the lowest value compared to C and P. These findings suggest the use of legume flour to partially replace wheat to delay the staling process. The breakdown was significantly higher in W than in legume flour. The pretreatment resulted in no effect on this parameter. It was previously found that similar results between lentil flour and extruded cooked lentil flour and cooked carioca bean flour [26,31]. Furthermore, limited breakdown in legume flour indicated that the paste viscosity was stable through the analysis and had good heating and shear stress during the food processing [32]. Furthermore, the low breakdown may be attributed to the effect of pretreatment (P and N) on reducing the activity of alpha-amylase. The setback was higher in W compared to pretreated flour. C had a higher setback than P and N, which was consistent with the previous studies [8,26]. Low setback viscosity was found to be correlated to retarding the retrogradation of flour; thus, P and N can potentially contribute to limiting staling.

According to Farinograph results (Table 3), the wheat flour-based dough had the lowest water absorption value compared to the legume blends (C50, N50, and P50). Consistently, previous studies showed increased water absorption in composites made by wheat flour and milled legume flour due to legume proteins/fiber’s high water-holding capacities [8,33]. N50 and P50 had significantly higher water absorption than C. Possibly, the green pea flour pretreatments, i.e., hydrothermal and soaking, followed by high pressure, might have caused the formation of partially pre-gelatinized starch, denatured the protein faction, and/or altered the fiber fraction structure easing their interaction with water and their incorporation into the dough matrix [14,29]. The development time of W50 and N50 resulted in statistically similar outcomes, while C50 and P50 required significantly higher time for dough development. The long development time of the dough was correlated to poor quality of baking, limited machinability, and relaxed stretchable properties [34]. Stability time was drastically reduced compared to W, where C50 showed the lowest value. These differences can be attributed to gluten dilution effect and the presence of damaged starch, resulting in a weak protein network [10,35,36]. These results are in concordance with the previous studies underlying that adding legumes increased dough development time and decreased dough stability [37,38].

### 3.2. Fresh Composite Bread Quality

The effects of pretreatment (PT) and the level of addition (LA) of green pea flour on the quality of fresh bread are summarized in Table 4. PT and LA significantly influenced all the parameters, while their interaction affected all parameters except bread cohesiveness. LA had the highest effect on all parameters compared to PT and PT × LA.

**Figure 2:**
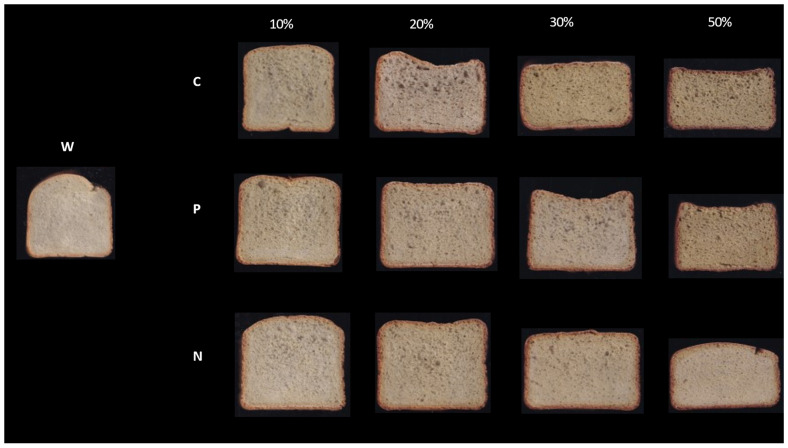
Pictures of the slices of the different loaves of bread.

Fresh bread properties are shown in Figure 2 and Table 5. Irrespective of the type of green pea pretreatment, bread made with a 10% addition level resulted in being statistically similar to the control bread. These results are consistent with the previous studies showing that adding 5–10% of different types of legume flour (e.g., lentil, chickpea, and bean) did not alter bread volume [29,39,40]. For level ≥ 20%, the increased LA reduced the specific volume. This can be explained by the gluten dilution effect, which resulted in weakening the protein network and, thus, reduced its ability to retain gases in the dough system. Furthermore, green pea flour has significantly higher fiber content compared to wheat flour (Table S1), which may disrupt the gluten network [21,39].

Proportionally to the level of wheat flour substitution, enriched bread showed significantly higher moisture content than the control. This can be attributed to the composition of pea flour enabling more water-holding capacity compared to wheat flour [17,41]. Overall, legume flour was reported to have a higher water-holding capacity compared to wheat flour [4,8]. Furthermore, the amount of added water to each formulation increased with the increase in green pea flour, which resulted in higher water content in the initial bread. Indeed, the increased total protein and pentosan contents in legumes were found to be associated with their high water-holding capacity [42]. With respect to the pretreatment effect, no patterns were observed among the different types of flour.

The crumb firmness of C10 and P10 was found to be similar to the control. This aligns with a previous study showing that roasted yellow pea flour at 10% did not cause any change in bread texture [14]. Beyond the 10% addition level, green pea addition resulted in firmer bread (up to eight times in the case of C50 and N50 compared to the control). It was reported that the high-level inclusion of legumes increased stiffness and resulted in a compact texture and low volumes and, thus, reduced bread softness [10]. Regardless of the addition level, bread made with the pretreatment P showed less increase in firmness compared to N and C.

Regardless of the pretreatment, cohesiveness was found statistically unchanged at up to a 10% addition level. Then, it was gradually reduced with the addition of conventional green peas flour, but it remained statistically similar to the control, up to 30% of P and N. The decrease in cohesiveness can be explained by the reduction in gluten content and the increase in non-gluten proteins [40].

### 3.3. Changes in Composite Bread Quality during Storage

Crumb moisture content and textural property changes in bread samples during storage are shown in Table S2, while statistical evaluation of the results is in Table 6.

For the moisture content, the addition level had the most pronounced effect, followed by the pretreatment effect compared to the effect of storage. Moisture content did not follow a trend during the storage period, and it significantly fluctuated for all the formulations. However, previous studies showed that the addition of legumes decreased moistness loss during storage [33,43]. These differences might have been due to the formulation and/or the type of legume used. Overall, P preserved its moistness better, followed by N and C and then W. This can be associated with the high protein and fiber contents of P, N, and C resulting in higher water-holding capacity and water retention than W. Composite bread made with 50% of legume flour showed the highest moistness by the end of storage period. Therefore, the use of pretreated green pea flour can delay staling by limiting water loss.

Crumb firmness was chiefly affected by the level of the addition and, to a lesser extent, by the storage period, while pretreatment had a minimal but significant effect. At 10%, all types of flour showed small fluctuations but no significant increase after 7 days. Probably, this result was due to starch retrogradation. It was observed that low levels of legume addition (5%) did not reveal a significant difference in the bread hardness [33]. This may suggest that incorporating legume flour at a low level (<10%) delayed the hardening of bread, as previously reported in bread made with 15% of yellow pea flour [33]. For 20% and 30%, the firmness of C20 and N20 significantly increased, but to a lesser extent in control. P20 and P30 showed a significant reduction in firmness after 3 days. The firmness of N50 increased during storage and resulted in the highest value after 7 days. Probably, at a high level of substitution, the starch retrogradation value increased, which increased crumb firmness. Additionally, legume polysaccharides and proteins contributed to the bread firmness [33]. However, firmness of C50 and P50 significantly decreased after 3 days. For P, we observed mold growth after 6 days of storage.

Cohesiveness is mainly governed by the storage time followed by the level of the addition, whereas the effect of the pretreatment was limited. Cohesiveness was reduced significantly during storage. The cohesiveness of W decreased faster than in green pea-enriched bread. This can be related to the high protein and fibers in green peas interfering with staling mechanisms, including moisture loss, starch retrogradation, and/or gluten network changes.

### 3.4. Nutritional Features of Composite Bread

**Figure 3:**
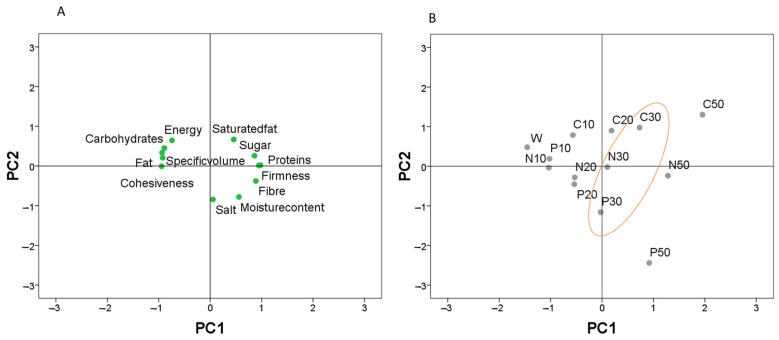
Principal components analysis of technological and nutritional characteristics of fresh bread enriched with pretreated pea flour. (**A**) biplot of the first two components. (**B**) rotated principal scores of bread samples. The orange circle shows the bread enriched with 30% having the central and positive positions meaning balanced specific volume and protein content (chosen as key features of discrimination).

The data in Table 7 shows that increased substitution of wheat with green pea flour caused, as expected, a progressive reduction in the product’s carbohydrate and fat contents as well as energy and a corresponding increase in proteins and fiber contents. This confirms the potential of legumes to improve the nutritional value of bread [44,45,46]. It is noteworthy that green pea flour substitution of ≥ 20% can lead to a product that can be claimed to be a “source of protein” in alignment with Regulation (EC) No 1924/2006, lastly amended by Regulation (EU) No 1047/2012. From an industrial perspective, the value of protein for products made with 20% substitution is borderline with the protein claim (>12%), and, thus, an enrichment of 30% is a safer choice to be sure that irrespectively to flour lot variability, protein content will be maintained. This hypothesis was confirmed through the use of principal component analysis (Figure 3). This analysis explained a total variability of 89%. The first component (PC1), accounting for 66% of the total variation, was expressed as a function of fat, carbohydrates, fiber, specific volume, proteins, cohesiveness, firmness, energy, and moisture content (Figure 3A), while the second component (PC2), accounting for 23%, was expressed as a function of salt and saturated fat. Based on this result, PC1 included the key criteria for bread quality, and, therefore, the bread samples projected into the factorial space (Figure 3B) can be discriminated. Bread (enriched with 30%) found in the central and positive positions is balanced in terms of specific volume and protein content (chosen as key features of discrimination). Extreme positive positions of PC1 are rich in protein but hard (bread with 50%), while extreme negative positions of PC1 have improved specific volume but low protein (bread with 10%). For bread with 20%, they were dispersed on both sides. Therefore, the best compromise between technological (firmness and volume) and nutritional considerations (protein claim) was the addition level of 30%.

The total starch content and in vitro starch digestibility (Table 8) were evaluated exclusively for 30% green pea-enriched bread. The addition of green pea flour reduced the total starch content compared to the control bread, while no significant differences were observed among the different pretreatments. W and C had similar RS values, followed by P and N. This indicated that adding pretreated green pea flour did not result in a relevant increase in RS. W had the highest RDS followed by the composite bread samples, which did not differ from each other. Legumes were reported to have starch entrapped in protective cell walls, limiting the access of digestive enzymes [4,47,48]. SDS was found higher in P due to their high pre-gelatinized starch favoring digestibility, followed by W, N, and C. A different behavior was observed in P30, as it showed the lowest RS but the highest SDS. The rates of digestibility (SDI) of W, P, and N were found to be the highest and statistically similar to each other compared to C30, having the lowest SDI. This can be correlated to the gelatinized starch in P and N, which was reported to be more digestible than untreated starch [47,48]. Conventionally milled flour showed the lowest SDI due to likely more extensive structural integrity of flour components (i.e., cell integrity, starch, proteins, fibers) due to the milder pre-processing step, thus leading to a limited starch digestibility [4,49].

## 4. Conclusions

In conclusion, the addition of pretreated green pea flour induced relevant changes in composite dough and bread properties. Flour pasting properties suggested that pretreated flour had high stability and lower retrogradation. It also showed higher water absorption, development time, and lower stability than wheat flour. By using 10% pretreated (P and N) flour, bread quality resulted in being similar to the control in terms of specific volume and textural properties, while increasing the inclusion level resulted in harder and more compact bread. During storage, bread made with legume flour at a low level (<10%) preserved its moistness. Nutritionally, increasing legume inclusion increased proteins and fiber. Based on PCA, the best compromise was the use of a 30% addition level to have balanced nutritional and technological quality. The use of 30% of milled legumes reduced the starch digestibility. P30 showed the lowest RS and the highest SDS. Overall, P and N can enable the production of soft and more stable bread.

## Figures and Tables

**Table 1:** Bread formulations with different levels of pretreated green pea flour.

Ingredients (%)	W	C10	C20	C30	C50	P10	P20	P30	P50	N10	N20	N30	N50
Flour	100												
W	100	90	80	70	50	90	80	70	50	90	80	70	50
C	-	10	20	30	50	-	-	-	-	-	-	-	-
P	-	-	-	-	-	10	20	30	50	-	-	-	-
N	-	-	-	-	-	-	-	-	-	10	20	30	50
Water *	55	57	59	61	64	60	65	69	78	58	61	64	69
Sugar	1.3												
Salt	2												
Yeast	2												
Olive oil	3.3												

* Water measured with Brabender farinograph to reach dough with consistency of 500 BU.

**Table 2:** Pasting properties of control wheat flour (W), conventional (C), new gen (N), and pre-gelatinized (P) green pea flour tested using a Micro-Visco-Amylograph.

	Initial Viscosity (BU)	Begining of Gelatinization Temperature (°C)	Peak Viscosity (BU)	Peak Viscosity Temperature (°C)	Cooling Maximum Viscosity (BU)	Breakdown (BU)	Setback (BU)
W	12.5 ± 0.5 ab	61.2 ± 0.5 b	648.3 ± 158.0 a	90.5 ± 0.5 b	911.0 ± 45.0 a	365.5 ± 59.0 a	535.0 ± 49.0 a
C	12.7 ± 0.6 a	69.8 ± 0.9 a	327.0 ± 23.1 b	95.2 ± 0.6 a	588.7 ± 52.0 b	47.3 ± 27.9 b	309.0 ± 57.7 b
P	11.0 ± 0.0 c	69.5 ± 0.2 a	281.0 ± 4.0 b	96.3 ± 1.4 a	518.0 ± 21.8 b	16.3 ± 11.0 b	217.0 ± 28.0 c
N	11.7 ± 0.6 bc	69.1 ± 0.4 a	317.3 ± 13.1 b	96.3 ± 0.9 a	397.0 ± 16.4 c	22.7 ± 3.1 b	136.3 ± 15.0 c

Different letters in the same column indicate significant (*p* < 0.05) differences between samples for the given parameter.

**Table 3:** Dough properties.

Doughs	Water Absorption (%)	Development Time (min)	Stability Time (min)
W	60.75 ± 0.35 d	1.50 ± 0.14 c	16.65 ± 0.07 a
C50	64.00 ± 0.70 c	8.80 ± 0.14 a	1.90 ± 0.28 d
P50	78.30 ± 0.21 a	6.85 ± 0.07 b	2.45 ± 0.07 c
N50	70.40 ± 0.07 b	1.70 ± 0.14 c	7.25 ± 0.07 b

Different letters in the same column indicate significant (*p* < 0.05) differences between samples for the given parameter.

**Table 4:** Multivariate analysis of the quality characteristics of fresh composite bread.

	PT		LA		PT × LA	
	SS%	Sig.	SS%	Sig.	SS%	Sig.
Specific volume	1.19	**	94.49	***	4.32	***
Moisture content	13.45	***	46.02	***	40.53	***
Firmness	6.94	***	86.74	*	6.32	***
Cohesiveness	25.82	***	68.55	***	5.64	ns

ns: not significant; *: *p* ≤ 0.05; **: *p* ≤ 0.01; ***: *p* ≤ 0.001; SS: sum of squares; sig: significance.

**Table 5:** Properties of fresh composite bread.

Bread	Specific Volume (cm^3^ g^−1^)	Moisture Content (g/100 g)	Firmness (N)	Cohesiveness (Dimensionless)
W	3.78 ± 0.03 a	40.96 ± 0.33 d	3.08 ± 0.94 h	0.67 ± 0.04 a
C10	3.85 ± 0.08 a	42.57 ± 0.06 c	3.09 ± 1.37 h	0.53 ± 0.72 abcd
C20	2.90 ± 0.11 c	42.73 ± 0.40 c	8.24 ± 1.06 e	0.48 ± 0.04 bcd
C30	2.59 ± 0.03 d	42.93 ± 0.24 c	13.62 ± 1.58 c	0.50 ± 0.02 bcd
C50	2.36 ± 0.10 e	43.19 ± 0.09 c	24.56 ± 2.68 a	0.39 ± 0.02 d
P10	3.75 ± 0.08 a	41.50 ± 0.23 d	3.08 ± 0.93 h	0.61 ± 0.07 ab
P20	3.33 ± 0.11 b	42.96 ± 0.13 c	5.89 ± 1.76 f	0.60 ± 0.04 abc
P30	2.79 ± 0.12 c	44.37 ± 0.82 b	7.24 ± 1.39 e	0.54 ± 0.04 abcd
P50	2.40 ± 0.05 e	47.90 ± 0.04 a	15.22 ± 1.95 b	0.44 ± 0.02 cd
N10	3.69 ± 0.10 a	43.00 ± 1.35 c	4.64 ± 1.12 fg	0.67 ± 0.03 a
N20	3.33 ± 0.12 b	43.47 ± 0.27 c	5.62 ± 1.46 f	0.61 ± 0.03 ab
N30	2.75 ± 0.13 c	43.20 ± 0.11 b	9.92 ± 1.23 e	0.55 ± 0.03 abcd
N50	2.05 ± 0.10 f	44.57 ± 0.14 b	24.12 ± 2.58 a	0.45 ± 0.02 cd

Different letters in the same column indicate significant (*p* < 0.05) differences between samples for the given parameter.

**Table 6:** Multivariate analysis of the quality characteristics of stored composite bread.

Factor		Moisture Content	Firmness	Cohesiveness
PT	SS%	31.01	2.55	1.75
	sig.	***	***	*
LA	SS%	44.64	72.30	12.68
	sig.	***	***	***
S	SS%	2.42	14.91	74.35
	sig.	***	***	***
PT × LA	SS%	14.00	1.19	0.38
	sig.	***	**	ns
PT × S	SS%	5.03	1.51	3.68
	sig.	***	***	***
LA × S	SS%	0.97	5.66	5.10
	sig.	ns	***	***
PT × LA × S	SS%	1.93	1.86	2.10
	sig.	ns	***	ns

ns: not significant; *: *p* ≤ 0.05; **: *p* ≤ 0.01; ***: *p* ≤ 0.001; SS: sum of squares; sig: significance.

**Table 7:** Nutritional composition based on Reg EU 1169/2011 of bread per 100 g.

Sample	W	C10	C20	C30	C50	P10	P20	P30	P50	N10	N20	N30	N50
Energy (Kcal)	218.36	210.68	208.35	205.88	201.49	214.87	208.02	199.72	185.99	212.46	206.68	206.48	198.95
Fat (g)	3.49	3.31	3.21	3.11	2.92	3.41	3.27	3.12	2.86	3.35	3.21	3.16	2.96
Saturated fat (g)	0.38	0.66	0.66	0.67	0.69	0.39	0.4	0.4	0.4	0.38	0.38	0.4	0.4
Carbohydrates (g)	42.59	40.06	38.57	37.06	34.16	40.93	38.65	36.17	31.88	40.42	38.32	37.26	33.91
Sugar (g)	1.63	1.65	1.71	1.77	1.89	1.68	1.7	1.7	1.72	1.64	1.64	1.69	1.72
Fibre (g)	1.01	1.31	1.62	1.94	2.57	1.41	1.76	2.09	2.69	1.4	1.77	2.18	2.91
Proteins (g)	5.52	6.18	6.96	7.74	9.29	6.11	6.59	6.97	7.73	6.15	6.74	7.5	8.72
Protein contribution to the energy value *	10.11	11.73	13.36 *	15.04 *	18.44 *	11.37	12.67 *	13.96 *	16.62 *	11.58	13.04 *	14.53 *	17.53 *
Salt (g)	1.09	1.06	1.06	1.05	1.05	1.27	1.43	1.57	1.83	1.06	1.04	1.05	1.02

Note: W = 100% wheat bread; P10 = 10% pre-gel; P20 = 20% pre-gel; P30 = 30% pre-gel; P50 = 50% pre-gel; N10 = 10% new gen; N20 = 20% new gen; N30 = 30% new gen; N50 = 50% new gen; C10 = 10% commercial; C20 = 20% commercial; C30 = 30% commercial C50 = 50% commercial. * “Source of protein” according to regulation (EC) No 1924/2006.

**Table 8:** Starch contents and digestibility.

Bread	TS g/100 g Dry Matter	RS g/100 g Dry Matter	RDS g/100 g dry Matter	SDS g/100 g Dry Matter	SDI (%)
W	71.39 ± 1.07 a	13.27 ± 0.27 a	54.34 ± 0.46 a	3.77 ± 0.40 b	76.11 ± 0.51 a
C30	55.03 ± 0.77 b	14.12 ± 0.64 a	39.10 ± 0.37 c	1.81 ± 0.29 c	71.05 ± 0.67 c
P30	55.55 ± 0.83 b	9.20 ± 0.71 c	41.04 ± 0.40 b	5.31 ± 0.58 a	73.87 ± 0.97 b
N30	54.80 ± 0.56 b	12.04 ± 0.78 b	40.95 ± 0.49 b	1.81 ± 0.15 c	74.72 ± 1.25 ab

RDS = rapidly digestible starch; SDS = slowly digestible starch; TS = total starch; RS = resistant starch; W = 100% wheat bread. Different letters in the same column indicate significant (*p* < 0.05) differences between samples for the given parameter.

## Data Availability

The data used to support the findings of this study can be made available by the corresponding author upon request.

## Supplementary Materials

The following supporting information can be downloaded at: https://www.mdpi.com/article/10.3390/foods12122284/s1, Table S1: Nutritional facts of wheat (W) flour and conventional (C), pre-cooked (P), and new-gen (N) green pea flour; Table S2: Changes in moisture content, firmness, and cohesiveness during storage.
