# Supplementary material for: Pretreated Green Pea Flour as Wheat Flour Substitutes in Composite Bread Making
Source: Foods. 2023 Jun 6;12(12):2284. doi: 10.3390/foods12122284 (PMC10297283; doi:10.3390/foods12122284)
Supplement: Supplementary file 1 [file foods-12-02284-s001.zip › foods-2376770-supplementary.pdf]

Table S1: Nutritional composition of pea flour by different pre-treatments.

	W	C	P	N
Energy (Kcal)	367	334	339	344
Fats (g)	3.3	1.7	2.4	2.1
Saturated fat (g)	0.2	0.3	0.4	0.4
Carbohydrates (g)	76.7	50.6	52.9	53.0
Sugars (g)	1.7	2.9	2.8	2.4
Fibers (g)	1.7	7.8	9.2	9.4
Proteins (g)	10.0	25.2	21.9	23.9
Salt (g)	0.01	0.01	0.01	0.01

Note: W= control wheat flour, C= conventional, P= Pre-cooked, N= soaked under pressure-streamed.

Table S2: Changes in moisture content, firmness, and cohesiveness during storage

	0	1	3	7
**Moisture content**				
**W**	40.96±0.33a	41.13±0.13a	40.87±0.29a	41.94±1.15a
**C10**	42.57±0.06a	42.32±0.29ab	41.9±0.25b	40.98±0.29c
**C20**	42.73±0.4ab	43.46±0.12a	42.55±0.04b	40.85±0.35c
**C30**	42.93±0.24a	43.51±0.3a	42.36±0.39a	40.94±0.44b
**C50**	43.19±0.09bc	44.47±0.07a	43.6±0.6ab	42.34±0.17c
**P10**	41.5±0.23c	43.19±0.36a	42.37±0.36bc	42.89±0.21ab
**P20**	42.96±0.13ab	44.8±0.16a	43.69±0.57bc	44.2±0.22ab
**P30**	44.37±0.82b	46.1±0.14a	45.51±0.31ab	45.38±0.18ab
**P50**	47.9±0.04b	49.28±0.17a	48.42±0.4a	47.53±0.48b
**N10**	43±1.35a	42.15±0.12a	42.09±0.14a	41.82±0.39a
**N20**	43.47±0.27a	42.56±0.01a	43.29±0.12a	42.78±0.05a
**N30**	43.2±0.11a	43.33±0.12a	43.71±0.17a	43.31±0.31a
**N50**	44.62±0.13a	46.02±0.04a	44.93±0.19a	44.5±3.66a
**Firmness**				
**W**	2.81±0.91c	2.66±1.41d	6.39±1.85b	7.35±3.54a
**C10**	3.21±1.33c	6.39±1.85b	7.45±1.59a	10.07±5.29ab
**C20**	8.16±1.03d	13.87±2.27c	16.32±5.23b	22.72±3.82a
**C30**	13.62±1.53d	19.11±3.52b	27.39±5.99b	35.64±4.36a
**C50**	24.34±3.52d	38.20±4.44a	39.54±5.76a	33.66±6.42b
**P10**	2.77±0.9c	5.07±1.93d	17.92±6.96a	8.41±3.13ab
**P20**	5.65±1.71d	10.96±2.04c	19.60±4.25a	9.69±4.12b
**P30**	7.14±1.34d	17.68±3.15b	22.37±3.87a	11.50±3.06c
**P50**	15.11±1.89c	38.04±3.65a	32.91±2.94b	12.29±2.82d
**N10**	4.23±1.09c	3.74±1.37c	7.82±2.75b	9.51±2.54a
**N20**	5.32±1.44d	8.86±3.07c	15.88±2.64b	20.23±4.29a
**N30**	9.72±1.18c	20.48±3.24b	20.65±3.19b	30.13±4.93a
**N50**	24.34±2.49d	32.01±2.67b	32.89±3.87b	44.52±6.61a
**Cohesiveness**				
**W**	0.66±0.04a	0.51±0.05b	0.42±0.02c	0.42±0.02c
**C10**	0.51±0.05a	0.47±0.03b	0.36±0.01c	0.35±0.04c
**C20**	0.48±0.03a	0.41±0.04ab	0.31±0.03bc	0.27±0.02c
**C30**	0.49±0.02a	0.33±0.02b	0.28±0.04c	0.28±0.04c
**C50**	0.39±0.02a	0.29±0.02b	0.28±0.02b	0.26±0.03b
**P10**	0.61±0.06a	0.43±0.03b	0.37±0.02c	0.31±0.04c
**P20**	0.59±0.03a	0.36±0.02b	0.31±0.01c	0.27±0.03c
**P30**	0.54±0.03a	0.35±0.02b	0.32±0.02b	0.27±0.02c
**P50**	0.44±0.02a	0.31±0.02b	0.33±0.04b	0.27±0.03b
**N10**	0.66±0.02a	0.46±0.02 b	0.27±0.03c	0.35±0.02c
**N20**	0.61±0.02a	0.40±0.04b	0.35±0.03b	0.29±0.02
**N30**	0.54±0.03a	0.35±0.03b	0.32±0.049b	0.28±0.02b
**N50**	0.44±0.01a	0.36±0.03b	0.34±0.03b	0.27±0.03c
